# Shared Decision Making: The 9-Item Shared Decision Making Questionnaire Does Not Discriminate Between Surgeons

**DOI:** 10.7759/cureus.14274

**Published:** 2021-04-03

**Authors:** Alyssa Reese, Tyler Wanstreet, Sarah Callaham, Michele M Carr

**Affiliations:** 1 Otolaryngology, Jacobs School of Medicine and Biomedical Sciences, University at Buffalo, Buffalo, USA; 2 Medicine, West Virginia University School of Medicine, Morgantown, USA; 3 Otolaryngology, West Virginia University School of Medicine, Morgantown, USA

**Keywords:** sdm-q-9, shared decision making, otolaryngology

## Abstract

Purpose

To determine if shared decision making (SDM) scores vary between individual otolaryngologists in a large specialty clinic.

Methods

Consecutive patients that consented to surgery were surveyed using the 9-item Shared Decision Making Questionnaire (SDM-Q-9), a validated scale for SDM. Demographic details included the respondent's age, gender, education level, marital status, whether the consent was for themselves or their child, whether surgery was for malignancy, and surgery being performed. Scores were evaluated for all demographic variables, as well as individual surgeons, surgeons' gender, age category, and subspecialty.

Results

A total of 233 patients completed the surveys. No significant differences were found among individual and total scores for SDM when compared among or between patient demographics (p > 0.05). A total of 10 surgeons for whom five or more SDM-Q-9s were completed were included in the study. No significant difference was found when SDM was evaluated for surgeon characteristics as well (p > 0.05).

Conclusion

SDM scores do not vary between these otolaryngologists.

## Introduction

As scientific developments lead to new treatment options, patients and clinicians are able to explore several possibilities for disease management. However, the presence of numerous options also offers a source of discussion and often a dilemma when no treatment is clearly favored over another. In this case, shared decision making (SDM) offers a way to make a patient-centered choice [1]. 

SDM is a process that occurs between a patient and their clinician, as both parties focus on deciding on management best suited to the patient. The clinician acts as a guide and offers the relevant medical information, while the patient has the freedom to share their views pertaining to the discussed pros and cons of the treatment options [2]. Ultimately, the physician is able to synthesize the patient’s opinions in order to formulate a final recommendation. 

Beneficial outcomes of SDM have been discussed in the literature, including increased patient satisfaction and adherence to medication regimens [2-6]. A decrease in undesired care has also been identified with this method, as the patient’s choice is the main focus [2-6]. Thus, as clinicians move towards SDM, understanding factors that influence the practice of this method and its effectiveness is of importance for proper implementation. 

The purpose of this study was to determine if SDM, as perceived by the patient and measured by the 9-Item Shared Decision Making Questionnaire (SDM-Q-9), varied among surgeons in a practice group with a range of ages, genders, and subspecialties.

## Materials and methods

This study was conducted at a large, academic tertiary facility following West Virginia University Institutional Review Board approval. Participants who provided consent for surgery within Otolaryngology clinics were recruited from July 2018 through August 2019. Each patient or the patient’s parent/guardian then consented to study participation. This included the SDM-Q-9 (5-point Likert scale) patient version and a demographic questionnaire. The SDM-Q-9 was developed in order to evaluate SDM from the perspective of the patient and the physician, and has been validated with good acceptance, feasibility, and reliability [7-8]. Surgeons varied according to age category (younger, middle-aged, and older), gender, and subspecialty (general otolaryngology, pediatric otolaryngology, and head and neck surgery). Statistical analysis was performed with IBM Statistical Package for the Social Sciences (SPSS) 27.0 (2020, IBM Corp., Armonk, NY). The Kruskal-Wallis and Mann-Whitney tests of significance were performed for comparison of non-parametric data with a level of significance of p < 0.05. Pearson’s chi-square test was used for binary data. Power analysis revealed that for an expected effect size of 0.3, a moderate effect, and an alpha of 0.05, the power to detect a difference was 96%.

## Results

**Figure 1:**
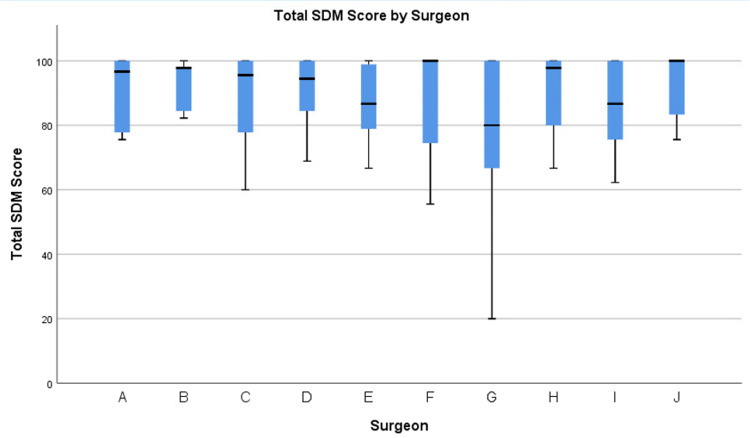
Total SDM-Q-9 score by the surgeon. SDM: Shared decision making; SDM-Q-9:9-Item Shared Decision Making Questionnaire.

A total of 10 surgeons for whom five or more SDM-Q-9s were completed were included in the study. A total of 233 patients participated. Demographic information appears in Table 1. Each respondent completed an SDM-Q-9 during the clinic visit where they participated in the consent process for surgery. The mean SDM-Q-9 score was 85.7 (95% CI: 83.1-88.4). Based on the aggregate data, there were no significant differences revealed among individual and total SDM scores (p > 0.05). A simple box plot of the scores can be found in Figure 1. 

**Table 1:** Mean SDM scores based on participant descriptive characteristics (no significant differences; p > 0.05). SDM: Shared decision making.

	Demographics	N (%)	Mean SDM Scores	P-value
Patient Identity N = 232	Self	118 (50.6)	84.5 (80.4-88.6)	> 0.05
	Parent of patient	114 (48.9)	86.9 (83.4-90.4)	
	Unknown	1 (0.4)	--	
Gender N = 233	Female	171 (73.4)	85.4 (82.2-88.5)	> 0.05
	Male	62 (26.6)	86.8 (81.6-91.9)	
Marital Status N = 233	Single	49 (21.0)	84.9 (78.3-91.5)	> 0.05
	Married	132 (56.7)	84.5 (80.8-88.3)	
	Separated/Divorced	23 (9.9)	83.7 (74.8-92.5)	
	Undisclosed	29 (12.4)	--	
Education N = 233	Not a college graduate	88 (37.8)	84.3 (79.5-89.0)	> 0.05
	College graduate	145 (62.2)	86.6 (83.4-89.8)	
Age (years) N = 233	18-29	54 (23.2)	87.3 (82.8-91.7)	> 0.05
	30-39	74 (31.8)	84.7 (79.4-90.1)	
	40-49	45 (19.3)	88.0 (82.1-93.9)	
	50-59	27 (11.6)	84.0 (76.1-91.9)	
	60 and older	33 (14.2)	83.8 (75.2-92.3)	
Previous Otolaryngologic Surgery N = 229	Yes	78 (34.0)	88.5 (85.3-91.8)	> 0.05
	No	151 (65.0)	84.0 (80.2-87.8)	
Pathologic Process N = 233	Benign	208 (89.3)	85.9 (83.2-88.7)	> 0.05
	Malignant	25 (10.9)	82.3 (67.6-96.9)	

SDM scores were also evaluated based on a variety of surgeon characteristics. Firstly, surgeons were separated into three categories based on age: under age 40, age 40-59, and over age 60. Non-parametric analyses revealed no significant differences between the groups (p > 0.05). Differences in SDM scores between female and male surgeons were also not found to be significantly different (p > 0.05). 

**Figure 2:**
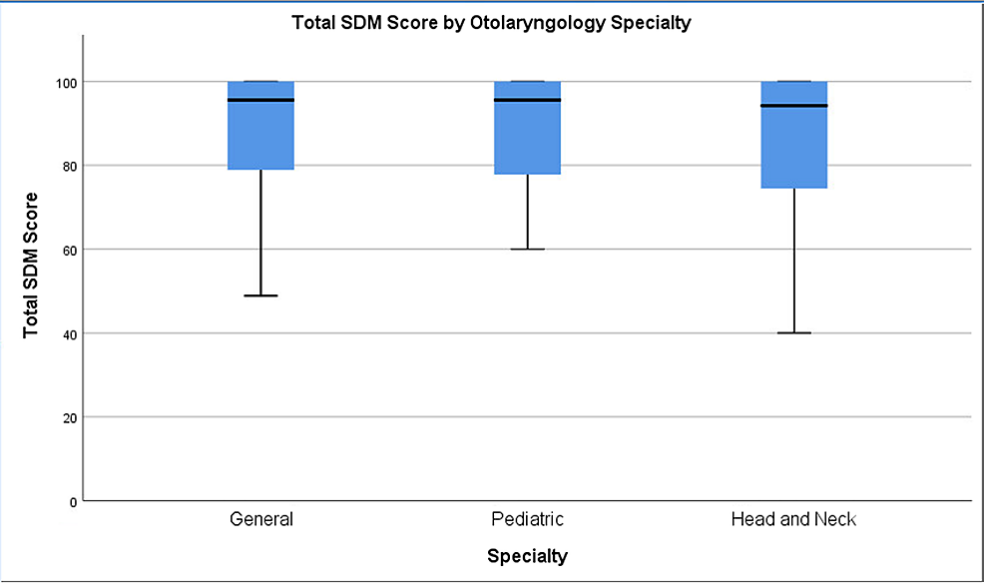
Total shared decision-making score by Otolaryngology specialty. SDM: Shared decision making.

The surgeons who participated in the study classified themselves as three different types of otolaryngology subspecialists. These included general otolaryngology, pediatric otolaryngology, and head and neck surgery. No significant differences were found between the SDM scores of the three different subspecialties (p > 0.05). A simple box plot of the SDM scores of each specialty can be found in Figure 2.

Non-parametric analyses were performed and revealed no significant differences among individual and total scores on the SDM-Q-9 when compared for respondent identity, age, gender, marital status, or education (p > 0.05). Additionally, individual and total scores on the SDM-Q-9 did not differ to a significant extent in terms of the pathologic process of the patient’s condition, or the patient’s prior history of Ear Nose Throat (ENT) surgery.

## Discussion

In this study, we discuss the association between SDM-Q-9 scores and a variety of patient and surgeon characteristics. Despite a large number of participants, we found no significant relationship between SDM-Q-9 scores and gender, marital status, age, education, the pathologic process of the patient’s condition, or the patient’s prior history of otolaryngology surgery. No significant relationship was also found between SDM-Q-9 scores and the gender, age, or subspecialty of otolaryngologists. 

The SDM-Q-9 has been validated with good acceptance, feasibility, and reliability [7-8]. Nevertheless, the data from this study suggest a possible ceiling effect of the SDM-Q-9. A high mean SDM-Q-9 score was obtained for all studied variables, similar to prior literature that has discussed this notion. Simon *et al.* acknowledged the prospect of a ceiling effect with the original SDM questionnaire and consequently, the SDM-Q-9 version attempted to adjust for this possibility through the inclusion of additional rating possibilities [7-8]. Despite the augmentation, a ceiling effect was still discovered by Calderon *et al.*’s study of the SDM-Q-9 in oncology practice [9]. Both Simon *et al. *and Calderon *et al*. attribute this ceiling effect to social desirability and the patient’s perceived need to appease the physician [7,9]. The weak correlations between the SDM-Q-9 and OPTION scales, another scale that evaluates the patient’s involvement in decision making, also supports the existence of the ceiling effect [10]. Overall, the ceiling effect that was observed in our data provides evidence that the SDM-Q-9 is not discriminatory in nature. This limits its ability to distinguish SDM practice habits between surgeons and evaluate their effectiveness. 

The absence of significant differences between and among patient and surgeon characteristics provides evidence that some factors that influence the physician-patient relationship during SDM are not controllable. If SDM-Q-9 scores were related to what surgeons do while discussing options or their characteristics, we would expect different surgeons to have different scores, as each has a typical way of managing patients. Since this was not the case, it offers the possibility that other factors that influence the physician-patient relationship may play a greater role than measurable characteristics - for example, the patient’s individual way of perceiving the world. All surgeons have had the experience of explaining the risks of a common surgery the same way to two different people and having their questions and concerns be completely different. However, it is also noteworthy that the lack of significant differences between the surgeons’ scores may also be due to the inherent characteristics of the SDM-Q-9. 

Differences in patient characteristics, surgery type, and pathology of the disease process also did not induce significant differences in SDM scores. This suggests that these characteristics do not play a significant role in SDM. However, this notion contrasts studies that have found an influence of education and disease process on SDM scores. Chang *et al*.’s study on the influence of SDM in cancer patients found that higher education level was related to higher health literacy and significantly correlated to higher SDM scores [11]. Additionally, through qualitative expert interviews, another study conducted by Müller-Engelmann found that patients, general practitioners, and health administration and research professionals believed that SDM was more appropriate in situations where the disease process was more severe [12]. Our results did not corroborate these two studies, but instead suggest that the patient’s perception of SDM can be equal for all education levels and is not impacted by the severity of the disease process being managed. 

SDM has been discussed as the ideal approach to discussing equally weighted treatment options with a patient by both physicians and patients alike [2]. However, despite the benefits, SDM is not used as frequently within surgical practice [2]. This study does not provide support for surgeons utilizing the SDM approach with their patients, as this was not our goal. However, it does suggest that the SDM-Q-9 may not be discriminatory enough to distinguish between SDM interventions, as a high ceiling effect was observed. Therefore, this study indicates that a more complex version of the SDM-Q-9 or a new SDM questionnaire altogether may offer a greater understanding of the SDM process and the implications of its use in a variety of clinical settings. 

Limitations

This study looked at only three different characteristics of each surgeon. Therefore, the study of additional factors such as race and ethnicity of surgeon and patient could be a potential focus of future research. We did not examine whether this encounter was the first between patient and physician. We did not have the physicians score the encounters. Our study also did not address the timing of the encounter, which has been shown to play a role in physician-patient communication in past research [13]. Our study is also based on an assumption that the surgeons generally approach the consent process in the same way with most patients. Furthermore, there were residents involved in these clinics, and their role in the patient’s perception of their experience was not evaluated.

## Conclusions

Despite a large number of patients and a diverse group of surgeons, this study found no association between SDM-Q-9 scores and individual surgeons, and scores showed a ceiling effect limiting their use at distinguishing SDM practice habits between surgeons. This suggests that differences in patient management and discussion of surgical options may not be reflected in SDM-Q-9 scores. Thus, some factors that impact SDM-Q-9 scores may not be controllable by the otolaryngologist.
